# Positive Youth Development and Internet Use in a Sample of Spanish Adolescents

**DOI:** 10.3389/fped.2022.842928

**Published:** 2022-05-23

**Authors:** Diego Gómez-Baya, Anna Jean Grasmeijer, Esther López-Bermúdez, Margarida Gaspar de Matos, Ramón Mendoza

**Affiliations:** ^1^Department of Social, Developmental and Educational Psychology, Universidad de Huelva, Huelva, Spain; ^2^Environmental Health Institute, Universidade de Lisboa, Lisbon, Portugal

**Keywords:** positive youth development, mental health, Internet use, social networks, videogames, adolescence

## Abstract

During the COVID-19 pandemic, the use of Internet in the adolescent population has increased. A growing research interest has been developed about the consequences of Internet use for adolescent development. Despite most studies have examined the impact of Internet use on some indicators of psychological maladjustment, few studies have addressed the detrimental impact on the positive indicators of mental health. Positive youth development (PYD) represents a positive view of adolescent transition to adulthood which focuses on building the strengths that make young people more resistant to negative outcomes and more capable to choose a positive life direction. This study aimed to analyze the relationships between different aspects of Internet use and overall PYD in a sample of Spanish adolescents. To reach this aim, a sample of 1,038 adolescents (50.1% boys, *M* age = 14.19, *SD* = 1.38), enrolled in 14 high schools in the city of Huelva (Spain), filled in some self-report measures of PYD and Internet use and experience, such as the frequency of Internet use on weekdays or weekends, the different uses of Internet (i.e., social networks, playing online, reading, surfing or looking for information, playing or downloading music, and searching, selling, or buying products), and the subjective experience using the Internet (i.e., acknowledgment of spending too much time playing or in the networks, and being in a bad mood if they do not play or use the networks). Results of a hierarchical regression analysis showed that the more hours using Internet on weekdays, the less PYD. Moreover, the experience of feeling bad when not using the networks and spending too much time with online playing was related to lower PYD. However, the use of Internet for reading or looking for information had a positive association with PYD. These results suggest some implications for practice, such as the need to promote an adaptive Internet use, by providing a safe online context that encourages the acquisition of positive social values and life skills.

## Introduction

Internet use has been increased among adolescents and young people. Data from the study Health Behavior in School-Aged Children in 40 European countries have indicated that a growing percentage of adolescents reported intensive electronic media communication (1). Thus, the percentage of intensive electronic media communication was 27% in 11-year-old adolescents, 36% in 13-year-old adolescents, and 41% in 15-year-old adolescents. Regarding the preference for online communication, data from this same international study have shown that the percentage increased from 10% in the sample aged 11 years to 15% at 13 years and 17% at 15 years. Furthermore, a study conducted in seven European countries (i.e., Greece, Spain, Poland, Germany, Romania, the Netherlands, and Iceland) on Internet addictive behavior (2) concluded that 13.9% of the adolescents aged 14–17 years displayed dysfunctional Internet behavior (characterized by a loss of control over their Internet use), highlighting the use of Internet for social networking and gaming. During the COVID-19 pandemic, the use of Internet in the adolescent population has increased even more. Recent research on adolescents from India, Malaysia, Mexico, and UK showed an increase in the use of social media sites and streaming services (3). In a study with Turkish adolescents, more than a half used Internet for 3 or more hours a day during the pandemic (4). This work underlines that the duration of Internet use was related to the risk of addiction and the development of psychosocial problems in adolescence. In a study conducted with a representative sample of secondary education students in Spain (5), data have indicated that many leisure time activities in adolescents implicated Internet use, such as the use of cell phone, tablet, or computer (96.3%), social networks (90.9%), watching movies or series on the Internet (86.3%), or playing videogames (67.7%). This report indicates that 94.8% of the adolescents have a cell phone with Internet, and the mean age of access to this device is 10.96. Moreover, 90.8% of the sample states a daily Internet connection and 98.5% is registered in at least one social network.

A growing research interest has been developed in the consequences of Internet use for adolescent development. Some studies have focused on the individual effects of Internet use and its problematic use (6). Mills (7) reviewed the negative effects of Internet use on adolescent cognitive development, indicating some possible negative effects on memory, analytical thinking, multitasking, and processing of social cues. Furthermore, Valkenburg and Peter (8) described the social consequences of the Internet for adolescents, underlining the conditions for an optimal experience and avoiding some possible risks, such as flaming or cyberbullying. However, a problematic Internet use was found to be related to worse interpersonal relationships and more dysfunctional coping strategies, as detected by Milani et al. (9) in Italian adolescents. In a sample of adolescents from the USA, Liu et al. (10) indicated that problematic Internet use was related to substance use, depression, and aggression, in both boys and girls. In adolescents aged 11–16 years from 25 European countries, the excessive Internet use was associated with some negative experiences, such as somatic problems, depressive symptoms, interpersonal problems, anxiety, Attention Deficit Hyperactivity Disorder (ADHD), low self-control, risky online behavior, missed school lessons, school problems, or drunkenness (11). A longitudinal study in a sample of adolescents aged 12–15 years from the Netherlands (12) concluded that the use of instant messenger was related to compulsive Internet use, depression, and loneliness after a 6-month follow-up.

Literature to date has specially underlined the consequences for the adolescent mental health of Internet use. In the USA, a national survey pointed out that talking with strangers online and the intensity of Internet use for communication were associated with an increased risk for depressive symptoms in adolescence (13). In the UK, a problematic use of Internet was related to conduct problems, hyperactivity, negative impact on daily life activities, depression, and poorer physical health (14). In the Netherlands, Internet use for non-communication purposes predicted more depression and social anxiety (15). A 4-year longitudinal study in Australia (16) observed that, in both females and males, compulsive Internet use was associated with increased mental health problems. In a sample of Chinese adolescents, Tan et al. (17) reported a prevalence of 17.2% of problematic Internet use and concluded that this use was correlated with more depressive symptoms and sleep disturbance. In this line, in a 9-month longitudinal study in China, Lam and Peng (18) pointed out that adolescents who were initially free of mental health problems but used the Internet pathologically developed depression as a consequence.

Despite most studies have examined the impact of Internet use on some indicators of psychological maladjustment, few studies have addressed the detrimental impact on the positive indicators of mental health. Valkenburg and Peter (19) concluded that Internet communication with a stranger had a negative effect on life satisfaction in a sample of Dutch adolescents. In addition, in the Netherlands, van der Aa et al. (20) showed that compulsive Internet use totally mediated the effect of daily Internet use on self-esteem. Another construct in psychological wellbeing with growing research interest is PYD. Lerner et al. (21) presented PYD as the result of mutually beneficial relationships between the individual and the context, which is related to thriving indicators, such as mental health or social contribution. PYD represents a positive view of adolescent transition to adulthood, which focuses on adaptive regulations by building the strengths that make young people more resistant to negative outcomes and more capable to choose a positive life direction (22). Within the construct of PYD, five dimensions are included, namely, competence (i.e., a positive perception of efficacy in different domains), confidence (i.e., an overall positive self-worth), connection (i.e., having positive social relationships), character (i.e., having a sense of integrity and being respectful of the rules of culture and society), and caring (which refers to a sense of empathy and sympathy for others) (23). To date, only two studies have addressed the effect of Internet use on PYD. In Malaysia, Joorabchi et al. (24) pointed out that the relationship between problems in Internet usage and PYD was significant, underlining the importance of promoting an adaptive use of Internet to satisfy psychological needs. In a sample of Chinese high school students, Dou and Shek (25) showed that PYD is related, both concurrently and after a 1-year follow-up, to less symptoms of Internet addiction.

Thus, more research is needed to examine the associations between Internet use and PYD in other cultural contexts, such as Spain. The relationships between PYD and other variables regarding developmental assets, lifestyles, social engagement, and psychological adjustment have been explored in previous studies with Spanish youth (26, 27), but the associations with Internet use have not been analyzed yet. Additionally, a deeper analysis could be recommended in this regard, in order to explore the PYD's interrelations with the frequency of Internet use on weekdays vs. weekend, the different uses of Internet (e.g., social networks, playing online, reading, surfing or looking for information, playing or downloading music, and searching, selling, or buying products), and the subjective experience using Internet (e.g., acknowledgment of spending too much time playing or on the networks, and being in a bad mood if they do not play or use the networks). This study aimed to analyze the relationships between these different aspects of Internet use on overall PYD in a sample of Spanish adolescents. Based on previous literature on the detrimental effect of Internet problematic use on mental health and the scarce evidence on PYD, we hypothesized that PYD was positively related to a constructive use of Internet and negatively related to the experience of spending too much time or being in a bad mood when not able to connect.

## Methods

### Participants and Data Collection Procedure

A sample of 1,038 adolescents (50.1% boys, *M* age = 14.19, *SD* = 1.38) participated in this study. They were enrolled in 14 high schools (7 were public and 7 were private) in the city of Huelva (Spain). The 4 years of Compulsory Secondary Education were represented (first year: 25.4%, second year: 25.8%, third year: 26.3%, and fourth year: 22.4%). Concerning the parental educational level of the sample, 28.4% of the mothers and 23.7% of the fathers had university studies, while around 15% had primary studies. Some adolescents indicated that they did not know the level of education of their fathers (28.7%) and mothers (22.3%). Regarding nationality, 95.2% of the adolescent participants were Spanish, while this percentage was a bit lower in the case of parents (90.3% of the mothers and 90.6% of the fathers). The selected sample was representative of the students of Secondary Education in the city of Huelva. Huelva is located in the South West of Spain, with a population of around 150,000, and is the capital of a region with the same name included in Andalusia. The province of Huelva is located between Seville and Badajoz (Spain) and Portugal.

Data were collected in the fall of 2020. These data were part of the study EVESO on the positive development and lifestyles of the Secondary Education students in Huelva (28). For the purposes of this study, the sample anonymously and individually filled in some self-report measures of PYD and Internet use and experience, spending around 20 min in their classrooms during a normal class time. During data collection, a member of the research group and a teacher were in the classroom. All participants and their parents previously gave informed consent for the participation in the study. The study obtained the approval of the Ethics Committee of the Andalusian Department of Health and Families.

### Instrument

#### Positive Youth Development

We used the Positive Youth Development Short Form, developed by Geldhof et al. (29) and adapted to Spanish by Gomez-Baya et al. (26). The questionnaire contains 34 items, which assess Competence (a positive view of one's actions in different domains), Confidence (a sense of self-worth in general), Character (considered as respect for the rules of one's society and culture, and a sense of integrity), Connection (or positive relationships with others), and Caring (defined as developing sympathy and empathy for others). The indicators were assessed by following a 5-point Likert-type scale, namely, 1 = strongly disagree and 5 = strongly agree, 1 = not important and 5 = extremely important, or 1 = not at all like me and 5 = very much like me. After calculating each separate dimension, the overall PYD was calculated by using the mean score. This overall score was used for the analysis in this study. The scale presented excellent internal consistency reliability, with α =0.90. Good factorial validity was observed with one factor explaining 52.64% of the variance and an eigenvalue of 2.63.

#### Internet Use and Experience

Some questions to assess the frequency of Internet use, the type of activity on the Internet, and the experience of using the Internet were used. First, the participants were asked about the number of hours on weekdays and on the weekend that they used. The Internet (“how many hours do you use Internet on weekdays” and “how many hours do you use Internet on the weekend”), with six response options, namely, none, half an hour or less, about 1 h, 2–3 h, 4–5 h, and 6 or more hours. Second, different uses of Internet were separately assessed using the same response options. Specifically, adolescents were asked about the hours using social networks, playing online, reading, surfing or looking for information, playing or downloading music, and searching, selling, or buying products. These indicators of Internet use presented good factorial validity, χ^2^(10) = 517.35, *p* < 0.001, with one factor reaching high explained variance (eigenvalue = 2.00, % variance explained = 39.90). Third, four indicators of the experience on Internet using social networks and games were presented, namely, “I spend too much time on the networks,” “I'm in a bad mood if I don't use the networks,” “I spend too much time playing online,” and “I'm in a bad mood if I don't play online.” A 5-point Likert-type scale was presented, namely, totally agree, agree, indifferent, disagree, and totally disagree. Responses from these four indicators were recoded to indicate with higher scores a worse experience using the Internet. The exploratory factor analysis showed that two factors, χ^2^(6) = 805.75, *p* < 0.001, composed of the indicators of the experience using social networks (eigenvalue = 1.23, % variance explained = 32.05) and the indicators of playing (eigenvalue = 1.77, % variance explained = 44.23). Furthermore, concerning the reliability, the indicators used to assess both the use and the experience of Internet, showed an acceptable internal consistency (α = 0.69).

### Data Analysis Design

Descriptive statistics were calculated for study variables, concretely mean and standard deviation of the overall PYD score, and the percentage of frequency distribution in the variables of Internet use and experience. Then, Pearson bivariate zero-order correlations were performed between all study variables. Finally, a hierarchical regression analysis was conducted to explain overall PYD. In this analysis, demographics (e.g., gender and academic year) were introduced in the first step. In a second step, variables concerning hours of Internet use on weekdays and weekend, as well as the type of use (e.g., social networks, gaming, reading/surfing/looking for information, playing/downloading music, and searching/selling/buying products), were introduced. Finally, the experience of Internet use on social networks and games was included in the regression equation. In each step, *F* and R^2^ values were calculated, and *t* and β scores were presented for each indicator to explain overall PYD. The *post-hoc* power analyses were performed in each step of the regression analysis by using G^*^Power 3.1. Statistical significance at *p* < 0.05 was considered in these analyses, which were performed using SPSS 21.0.

## Results

### Descriptive Statistics

Table 1 presents the frequency distribution of the variables about the use of Internet. Results showed that 43.5% of the sample reported more than 4 h of Internet use on weekdays, and 30.4% of the sample reported around 2–3 h. On weekends, 72.9% reported more than 4 h of Internet use and 19.2% reported 2–3 h. Regarding the type of Internet use, the most frequent uses of Internet were the social networks, playing music, and games. Concerning social networks, 37.6% indicated more than 6 h a week, and 27.2% of the sample indicated 2–3 h. With regard to playing or downloading music, up to 28.9% showed a weekly use of more than 4 h, while 19.8% reported a frequency of 2–3 h. Concerning gaming frequency, 22.9% showed a weekly use of more than 4 h and 14.9% reported 2–3 h. The less frequent Internet uses were reading, surfing, or looking for information (31.5% reported half an hour or less and 26.3% about 1 h) and searching, selling, or buying products (20.6% indicated a use of half an hour or less and 10% about 1 h).

**Table 1:** Descriptive statistics of variables about the frequency of Internet use.

	**None**	**Half an hour or less**	**About 1 h**	**2–3 hs**	**4–5 h**	**6 or more hours**
Hours of internet use on weekdays	3.5	6.5	16.1	30.4	20.4	23.1
Hours of internet use on the weekend	1.1	1.6	5.8	19.2	27.9	44.4
Use of social networks	6.5	10.2	18.5	27.2	15.8	21.8
Playing online	36.4	11.9	13.8	14.9	7.5	15.4
Reading, surfing or looking for information	10.8	31.5	26.3	17.7	7.9	5.9
Playing or downloading music	10.6	18.8	21.9	19.8	13.4	15.5
Searching, selling or buying products	59.2	20.6	10.0	5.3	2.4	2.6

Table 2 shows the frequency distribution of the variables about the experience of Internet. Results pointed out that 43% acknowledged that they spent too much time on the networks and 25.3% indicated that they spent too much time regarding online gaming. Furthermore, 20.1% of the sample indicated that they were in a bad mood if they did not use the networks, compared to 15.3% who stated this about gaming. Furthermore, a notable mean score was observed in overall PYD, with a mean of 3.66 (SD =0.53), with a minimum of 1.44 and a maximum of 5.

**Table 2:** Descriptive statistics of variables about the experience of Internet use.

	**Totally agree**	**Agree**	**Indifferent**	**Disagree**	**Totally disagree**
I spend too much time on the networks	20.4	22.6	32.9	13.3	10.8
I'm in a bad mood if I don't use the networks	6.8	13.3	24.1	28.1	27.7
I spend too much time playing online	10.3	15.0	24.9	18.5	31.3
I'm in a bad mood if I don't play online	5.7	9.6	21.1	22.9	40.7

### Associations Between Internet Use/Experience and PYD

Table 3 presents the bivariate correlations between overall PYD and the variables about Internet use and Internet experience. The results showed that PYD had small negative associations with the hours of Internet use on weekdays and on weekends. No significant associations with PYD were observed by the different uses of Internet, except for a small positive correlation with the use for reading, surfing, or looking for information. Furthermore, negative correlations were observed between PYD and the variables of Internet experience. Thus, less PYD was related to spending too much time on social networks or games, with being in a bad mood if they did not use the networks or play online.

**Table 3:** Bivariate Pearson correlations between PYD, Internet use, and Internet experience.

	**1**	**2**	**3**	**4**	**5**	**6**	**7**	**8**	**9**	**10**	**11**	**12**
1.-PYD	1											
2.-Hours of internet use on weekdays	-0.12***	1										
3.-Hours of internet use on the weekend	-0.08*	0.60***	1									
4.-Use of social networks	-0.06	0.51***	0.42***	1								
5.-Playing online	0.01	0.14***	0.25***	0.16***	1							
6.-Reading, surfing or looking for information	0.09**	0.14***	0.12***	0.20***	0.23***	1						
7.-Playing or downloading music	-0.01	0.30***	0.27***	0.40***	0.17***	0.32***	1					
8.- Searching, selling of buying products	0.02	0.12***	0.07*	0.20***	0.16***	0.25***	0.34***	1				
9.-I spend too much time on the networks.	-0.10**	0.21***	0.17***	0.39***	-0.06	0.01	0.16***	0.11**	1			
10.-I'm in a bad mood if I don't use the networks	-0.17***	0.18***	0.16***	0.29***	-0.04	-0.07*	0.09**	0.09**	0.40***	1		
11.-I spend too much time playing online.	-0.09**	-0.02	0.09**	-0.08*	0.43***	0.05	0.01	0.06	0.08**	0.08*	1	
12.-I'm in a bad mood if I don't play online	-0.11***	0.03	0.09**	0.04	0.35***	-04	0.01	0.08*	0.01	0.27***	0.63***	1

PYD: Positive youth development. ***p < 0.001; **p < 0.01; *p < 0.05.

Regarding the associations between the Internet variables, we found positive interrelations between the frequency of use on weekdays and on weekends, with the different Internet uses. Finally, moderate positive associations were observed between the number of hours spent on social networks, and the acknowledgment of spending too much time or being in a bad mood when they did not use them. In the same line, the frequency of gaming was positively associated with the acknowledgment of spending too much time playing and being in a bad mood if they did not play.

### Hierarchical Regression Analysis

Table 4 describes the results of the hierarchical regression analysis to explain PYD on the basis of demographics, the use of Internet, and the subjective experience using Internet. Results indicated that demographics simply explained 1% of the variance, with the academic year presenting a negative effect on PYD. Less PYD was found in the third and fourth years of Secondary Education, compared to the first two courses. In the second step of the analysis, a 3.5% variance was achieved based on the negative effect of the hours of Internet use on weekdays and the positive effect of the use of Internet for reading, surfing, or looking for information. Finally, the total model reached a 7% variance of PYD after including the variables of Internet experience. Spending too much time playing online and being in a bad mood if they did not use social networks had a negative effect on PYD. These variables about Internet experience provided the greatest increase in the explained variance of PYD.

**Table 4:** Hierarchical regression analysis of the effects by demographics, variables of Internet use, and Internet experience on PYD.

	***F***	**R^**2**^**	**ΔR^**2**^ / Power**	***t***	**β**
**Step 1: Demographic**s	4.42*	0.010	0.010 /0.773		
Gender				−1.60	−0.07
Academic Year				−3.05	−0.11**
**Step 2: Internet use**	3.57***	0.035	0.025 /0.959		
Hours of internet use on weekdays				−2.14	−0.09*
Hours of internet use on the weekend				−0.23	−0.01
Use of social networks				0.55	0.02
Playing online				0.51	0.02
Reading, surfing or looking for information				3.00	0.11**
Playing or downloading music				0.31	0.01
Searching, selling of buying products				1.00	0.04
**Step 3: Internet experience**	5.13***	0.070	0.035 /0.998		
I spend too much time on the networks.				0.21	0.01
I'm in a bad mood if I don't use the networks				−3.37	−0.13**
I spend too much time playing online.				−1.98	−0.09*
I'm in a bad mood if I don't play online				−1.22	−0.06

Dependent variable: Positive Youth Development (PYD). ^***^p < 0.001; ^**^p < 0.01; ^*^p < 0.05.

## Discussion

This study aimed to examine the relationships between overall PYD and different aspects of Internet use, i.e., the hours on weekdays or weekends, the differential effects of the type of Internet use, and the subjective experience using Internet, in a sample of Spanish adolescents. The results showed that more PYD was observed in adolescents with less Internet hours on weekdays. Moreover, those participants who were in a bad mood if they could not use the networks or those who acknowledged that they spent too much time playing online reported less PYD. These results are in line with our hypothesis, which established that PYD would be related to less problematic Internet use. This result is also in line with evidence from Chinese adolescents in the work described by Dou and Shek (25). Furthermore, we have observed that the use of Internet for reading or looking for information was positively associated with PYD. This result highlights that the Internet could have positive implications for PYD if an adaptive use is promoted in the adolescent population, as already concluded by Joorabchi et al. (24) in Malaysia.

The problematic use of Internet could limit or hinder some daily activities that may enrich adolescent health and well-being, such as physical activity, academic engagement, close interpersonal relationships with peers, or good family communication. If the pattern of Internet use does not allow for the development of self-confidence, self-efficacy, positive connection with others, positive social values, and empathic skills, it may be difficult for the development of PYD and mental health in adolescence. Thus, when adaptive use of Internet is promoted, characterized by self-control and autonomy, skills development, and complementary to offline interactions, and used for empowerment and social participation, it may nurture PYD. In line with Valkenburg and Peter (19), the online communication may lead to psychological well-being when it encourages online self-disclosure and good quality in social relationships. Talking with strangers or solitary forms of Internet use would be related to detrimental effects. Maladaptive Internet use is more likely to appear when previous mental health problems are reported (30) or when psychological needs are not adequately satisfied in offline contexts (31). However, a health-related Internet use by adolescents in order to seek information about healthcare and support systems may have positive implications (32). In addition, an educational use of Internet to promote distance learning and support virtual learning may have positive consequences (33). Internet may also be used for building a youth network that fosters social participation and action, by providing distance mentoring and training, as shown in the project Dream teens in Portugal (34).

Despite the interesting contributions of this study, some limitations should be considered. First, because a cross-sectional design has been followed, our conclusions can only be based on the bidirectional interrelations. To be able to establish relationships between antecedents and consequences, a longitudinal design is recommended, in line with the work described by Ciarrochi et al. (16), Dou and Shek (25), or van den Eijnden et al. (12). Second, a limitation may come from the use of self-reports, which provides subjective information about the Internet use and its experience, which may be biased by social desirability. This information could be complemented with more objective measures and other key informants, such as the parents. Third, some variables should be further examined in the interrelation between PYD and Internet use, such as mental health problems, offline experiences with peers, personality traits, or self-regulation skills (20, 30, 31, 35). Future research may also provide further examination of gender differences in the uses of Internet and their differential effects on wellbeing and PYD, as already pointed out by Ybarra et al. (13).

This study suggests some implications for practice and policy. Further policy regulation is needed to protect the adolescent use of Internet, in order to provide a safe online context that encourages the acquisition of positive social values and life skills, as performed in school or family, and develops healthy attitudes and psychological wellbeing (36). Psychoeducational programs may be recommended to foster self-regulation skills and critical thinking in the use of Internet (37, 38). School-based prevention programs should not only reduce the Internet time, but also provide skills development and the use of protective and harm-reducing factors, within a multirisk behavior intervention (39). Another approach of intervention could be focused on Internet addiction and other forms of risk behaviors, and could target vulnerable adolescents as well as their parents (40). Furthermore, PYD programs have been found to be effective for strengths development in the transition to adulthood, which in turn contributes to desirable outcomes, such as prosocial behavior and social skills, peer acceptance, school achievement, self-awareness, self-management, and responsible decision-making, among others (41). These consequences of PYD programs could also be beneficial for promoting a more adaptive and self-regulated Internet use.

In conclusion, this research has presented some evidence of the relationships between PYD and Internet use in a sample of Spanish adolescents. The results have indicated that the more hours using Internet on weekdays the less PYD, and the experience of feeling bad when not using the networks and spending too much time with online playing was related to lower PYD. Furthermore, the use of Internet for reading or looking for information had a positive effect on PYD. Thus, this study underlines the need to design intervention programs to promote a self-controlled and adaptive Internet use in adolescents, which may nurture PYD, e.g., through skills development, positive social relationships, and empowerment, instead of isolation, addictive behavior, and other mental health problems.

## Data Availability Statement

The raw data supporting the conclusions of this article will be made available by the authors, upon reasonable request.

## Ethics Statement

The research project was reviewed and approved by the Ethics Committee of the Andalusian Department of Health and Families (29th January 2020). Written informed consent to participate in this study was provided by the participants' legal guardian/next of kin.

## Author Contributions

All authors listed have made a substantial, direct, and intellectual contribution to the work and approved it for publication.

## Funding

This research received funding from the Council of Huelva under Grant number 27-2019, awarded to RM and DG-B.

## Conflict of Interest

The authors declare that the research was conducted in the absence of any commercial or financial relationships that could be construed as a potential conflict of interest.

## Publisher's Note

All claims expressed in this article are solely those of the authors and do not necessarily represent those of their affiliated organizations, or those of the publisher, the editors and the reviewers. Any product that may be evaluated in this article, or claim that may be made by its manufacturer, is not guaranteed or endorsed by the publisher.
